# Revisiting the relationship between attributional style and academic performance

**DOI:** 10.1111/jasp.12356

**Published:** 2015-10-06

**Authors:** Diane M. Houston

**Affiliations:** ^1^ Centre for the Study of Group Processes, School of PsychologyUniversity of KentCanterburyKentCT2 7NPUnited Kingdom

## Abstract

Previous research into the relationship between attributions and academic performance has produced contradictory findings that have not been resolved. The present research examines the role of specific dimensions of attributional style in predicting subsequent academic performance in a sample of pupils (*N* = 979) from both high‐ and low‐achieving schools. Hierarchical regression and moderation analyses indicate that internal, stable, and global, attributional styles for positive events predict higher levels of academic performance. Global attributions for negative events were related to poorer performance across all schools. Stable attributions for negative events were related to higher levels of performance in high‐achieving schools but not in low‐achieving schools. Higher levels of internality for negative events were associated with higher performance only in low achieving schools.

The aim of this paper is to test and develop theory regarding the relationship between attributional style and academic performance. Previous research has produced contradictory findings and many published studies have limitations in their specificity of measurement, small sample sizes, and differences in achievement context. This raises questions for theories of the role of attributions in academic achievement and the use of attributional retraining in improving achievement. The present research examines the role of attributional style, for positive and negative events, in predicting subsequent academic performance in a large sample of school students. It theorizes and tests the role of achievement context in the attribution–performance relationship.

## Attributional theory of achievement motivation

The term attribution refers to the causal inferences people make to predict and explain the behaviors of self and others (Heider, 1958). A considerable body of research has explored attributions following academic success or failure. This work consistently demonstrated that “self‐serving” attributions occur frequently in academic settings whereby people tend to attribute academic successes to internal and/or stable causes (e.g., ability, effort) and attribute academic failures to external and/or unstable causes (e.g., task difficulty, luck) (e.g., Frieze & Weiner, 1971; Miller & Ross, 1975).

Weiner's (1979, 1985, 1986) attributional theory of achievement motivation described how academic performance, expectations of future performance, and emotional reactions to performance, are all influenced by causal attributions. Weiner (1979) proposed that three causal dimensions are central to these processes. The first dimension, locus, distinguished between attributions about performance to internal versus external causes. The second dimension, stability, distinguished between attributions of performance to enduring versus variable causes. The third dimension, controllability, distinguished between attributions of performance to those which are within the individual's control versus those which are not. Weiner argued that those high in achievement motivation attribute success to high ability and effort, and failure to lack of effort, not lack of ability.

Weiner (1985) described a “fundamental psychological law relating perceived causal stability to expectancy change… . If an outcome of an event is ascribed to a stable cause, then that outcome will be anticipated with increased certainty or with an increased expectancy in the future.” Across a large number of studies, attribution of failure to stable causes was demonstrated to result in greater expectation of future failure.

## The reformulated model of helplessness and depression

The reformulated model of helplessness and depression (Abramson, Seligman, & Teasdale, 1978) and the hopelessness model of depression (Abramson, Metalsky, & Alloy, 1989) proposed that individual differences in styles of attribution predispose individuals to explain events in a consistent manner across different contexts and the lifespan. These differences in attributional style were hypothesized to determine whether an individual is at risk of developing cognitive, motivational, and emotional deficits associated with hopelessness and depression. Globality, the extent to which a cause generalizes across many situations, was included in the model as an alternative third attributional dimension to Weiner's (1979) concept of controllability and it is this concept that has been used in all research within the helplessness/hopelessness framework since 1978.

Within the models, the attribution of positive events to stable, global, and internal factors, and the attribution of negative events to external, unstable, and specific factors, is considered to be a “healthy” attributional style. The opposite style, particularly the attribution of negative events to internal, stable and global causes, is hypothesized to be “depressogenic” and to act as a diathesis that interacts with life events to produce depression (Abramson et al., 1989). It is worth noting that within the literature, some researchers also use the terms “pessimistic” and “optimistic” (e.g., Satterfield, Monahan, & Seligman, 1997) and that negative and positive life events are also often referred to as “failure” and “success” (e.g., Tiggemann & Crowley, 1993).

## Attributional style and academic performance

Research into the attribution–achievement relationship using the reformulated model of helplessness and depression reported that internal, stable, and global styles of attribution for negative events (also referred to as “pessimistic styles”) were associated with lower levels of academic achievement in children aged 8 to 11 years (Nolen‐Hoeksema, Girgus, & Seligman, 1986), undergraduate students (Peterson & Barrett, 1987), and a sample of life insurance salesmen (Seligman & Schulman, 1986). However, completely opposing findings have also been reported. Houston (1994) found that British students with stable, global attributional styles for negative events performed better than others on academic and ability‐related tasks across a series of three studies. Houston speculated that previous studies might have been confounded by the effects of depressed mood on performance. Satterfield et al. (1997) also found that postgraduate law students with pessimistic styles of attribution demonstrated higher levels of academic achievement. In a study of Australian university students, McKenzie and Schweitzer (2001) found that pessimistic attributional style was predictive of higher Grade Point Averages (GPAs) at the .10 probability level” (p. 30). In a study of North American students, Gibb, Zhu, Alloy, and Abramson (2002) reported that freshmen with pessimistic attributional styles (internal or stable attributional styles for negative events) received higher cumulative GPAs during college if they had high levels of ability (i.e., high Scholastic Aptitude Test (SAT) scores) than if they had low levels of ability (i.e., low SAT scores). Some published research has also reported finding no relationship between attributional style and academic performance (e.g., Bridges, 2001; Tiggeman & Crowley, 1993). Given the potential clinical and educational importance of potential interventions to influence attributional style it is clearly necessary to gain evidence that can help to explain these mixed findings.

## Academic ability and achievement context

Recent theorizing has explored the ability‐context of the attribution–performance relationship. Evidence that has supported the hypothesis that pessimistic attributional style should be related to poor performance has generally come from broad‐ability samples (schoolchildren, undergraduates, salesmen). Evidence for the opposing hypothesis has generally been from samples that represent academically selective contexts. As Houston's (1994) findings were from a sample that represented the top 10% of exam performance of British school leavers, she proposed that ability or achievement context may moderate the relationship between attributional style and academic performance. Consistent with this idea, Satterfield et al.'s (1997) evidence was from a sample of North American postgraduate law students who had already achieved high GPAs. Moreover, Gibb et al.'s (2002) North American sample was divided into those of higher and lower academic ability on the basis of their SAT scores and the relationship between pessimistic styles and higher performance was found only among those with higher academic ability.

## Measurement of attributional style

One source of variability in studies that examine the attribution–performance relationship within the helplessness/hopelessness framework is the way in which attributional style is measured. Some research has used a composite measure, averaging scores on internality, stability and globality dimensions (e.g., Peterson & Barrett, 1987). However, the internal–external dimension has been criticized (e.g., Miller, Smith, & Uleman, 1981). In two studies, White (1991) demonstrated that asking people to make causal attributions to the person (internal) or the situation (external) does not provide a clear distinction as such a dichotomy fails to capture the distinction between behavior that is conscious and intentional and behavior that is unconscious and unintentional. For example, one might attribute exam failure to lack of sleep (an internal cause) but this lack of sleep could be due to the decision to go to an all‐night party (intentional) or being woken by one's neighbor (unintentional). In addition, Joiner and Metalsky (1999) highlighted the relatively low reliability of the internality dimension of the attributional style questionnaire and suggested this might be a construct‐related issue, rather than measurement problem. A consequent shift of emphasis within the Hopelessness model of depression was to a focus on the stability and globality dimensions. A new composite of the stability and globality dimensions was proposed and termed *attributional generality* (Abramson et al., 1989), reflecting the extent to which causes of events are perceived to be stable and global versus unstable and situation specific. Researchers have continued to include the internality dimension in their studies, but not always as part of a composite measure (e.g., Gibb et al., 2002). Studies that have examined each of the attributional dimensions separately, rather than as a composite of all three dimensions, seem to have been more likely to report a positive association between attributional dimensions associated with helplessness/hopelessness and academic performance (e.g., Houston, 1994). In the present study and in line with Gibb et al. (2002), all attributional dimensions will be examined individually, as well as in composite form, in order to more clearly examine which best predict academic performance.

A further aspect of the attribution–performance relationship, which is relatively under‐researched, is the relationship between attributional style for *positive* events and academic performance; most published studies only report the relationship with attributional style for negative events.

## Sampling

Many of the studies reported above, whether they demonstrate a positive or negative relationship between attributional style and academic performance, have been conducted on small samples of less than 100 participants (exceptions being Satterfield et al., [*N* = 387] and McKenzie & Schweitzer [*N* = 197]). In addition, all of the studies have been conducted in only one class or institution/organization. Thus there is a possibility that the findings of previous studies are in part determined by the organizational culture or teaching style in particular institutions. The present research addresses this limitation by including a larger sample that spans a wide ability range, varied socioeconomic status, and a balanced gender mix.

## Hypotheses

The goal of the study reported in this paper is to provide a more comprehensive test of the relationship between attributional style and academic performance across a range of different schools which represent different achievement contexts. The study is prospective in design and involves a large sample of school students aged 15–16 years, in the academic year in which they take their first set of public examinations. Scores for each attributional dimension were examined separately as well as in composite, and style for both positive and negative events was measured.

### Helplessness hypothesis

The hypothesis that can be extended from the reformulated model of helplessness and depression (Abramson et al., 1978) is that internal, stable, and global styles of attribution for negative events should be related to lower levels of academic achievement and that internal, stable, and global styles for positive events should be related to higher levels of academic achievement.

### Context hypothesis

The hypothesis that follows from findings of Houston (1994) and Gibb et al. (2002) is that the relationship between attributional style and academic performance will differ according to academic ability/achievement context, such that stable attributions for negative events will be related to higher levels of academic achievement in high‐achieving schools, but not in lower achievement contexts.

## Method

### Participants

Participants were 979, 11th grade students drawn from ten secondary schools that spanned the full ability range from part of an Education Authority in the South East of the United Kingdom. Four of the schools were known to have a strong record of academic performance. These were two private schools and two grammar schools that select high‐ability pupils based on an Education Authority exam at the age of 11. The remaining six schools were comprehensive schools whose selection criteria did not include academic ability but was based on faith or area of residence. The mean age of the students was 15.33 years (SD = .49). They were in the school grade in which students are required to complete the General Certificate of Secondary Education (GCSE), which comprises a set of public examinations in each of up to 10 subject areas. The exams are graded by an independent national exam board and the grades are nationally accepted qualifications that serve as a basis for entry into further education and or employment selection. Across the measures, the listwise valid *N* was 948, and the smallest pairwise *N* was 979. Missing data showed no consistent pattern and was not correlated with gender or school type.

### Measures

#### Attributional style

A version of the ASQ (Peterson et al., 1982)/EAESQ (Metalsky, Halberstadt, & Abramson, 1987) was used to measure attributional style related to achievement‐related positive and negative life events. This measure comprised 12 hypothetical scenarios all relating to achievement, with six positive situations and six negative situations. Participants are instructed to imagine that each of the 12 hypothetical situations actually happened to them and to report the most likely cause. Using a 7‐point Likert scale, participants rated the cause on three dimensions: internal‐external, stable‐unstable, and global‐specific.

#### Academic performance

The actual academic performance of students was measured by using actual examination results, which were made available by the schools at the end of the study. Exam performance data were available for 979 of those who participated in the study. The total examination score for each student was calculated using the national points system for GCSE examinations employed by the UK educational system at the time of assessment. Grades can range from A* (8 points), A (7 points), B (6 points), C (5 points), D (4 points), E (3 points), F (2 points), and G (1 point).

### Procedure

The research was introduced as concerning social attitudes and experiences in education. Questionnaires were completed in class. Participants were asked to wait silently until all in the classroom had completed the measures. Anonymity and confidentiality was ensured by having participants generate a unique personal code number, and they returned questionnaires to the researcher in sealed unlabeled envelopes. Parental consent to participate was obtained from all those who were under 18 years. None declined. All students within the school year group participated other than those who were absent on the day of testing. Questionnaires were administered by a female researcher to students between January and March. In May/June, students completed the GCSE public examinations. The results of these public examinations were announced in August.

## Results

### School differences

The mean exam score across all participants was 51.74 (SD = 26.69). Analysis of Variance (ANOVA) to compare the performance levels of the 10 schools revealed two distinct subsets that were non‐overlapping. The four schools known for their strong academic record all attained significantly higher scores than all of the other schools (all *p*'s < .001), and did not differ among themselves (all *p*s > .21). Thus, the schools were classified, and for brevity labeled, as “high achieving” (mean GCSE level = 76.05, SD = 19.80) or “low achieving” (*M* = 39.49, SD = 20.63). The difference between these two means was highly significant, *F* (1, 978) = 740.19, *p* < .001, η^2^ = .42. For purposes of further analyses school type was coded as a binary variable (1 = high achieving, 2 = low achieving).

### Attributional style

Attributional style research has used internality, globality, and stability separately as well as using scores that combine stability and globality into an index of generality and a composite of all three dimensions score. Table 1 shows the descriptive statistics, reliability coefficients and correlations among each of the attributional dimensions and their relationships with performance. Attributional style for positive events and attributional style for negative events were analyzed separately in the present analyses. Specifically, we conducted separate tests of the effects of each dimension of attributional style and its interaction with school level. Follow‐up analyses were conducted with aggregated generality and composite measures. For economy of presentation, the results of the composite analyses are provided in Tables 2 and 3, and significant effects of separate dimensions of attributional style are reported in the text.

**Table 1:** Mean Scores, Reliability Analysis, and Intercorrelations for Attribution and Academic Performance

	Positive Internal	Positive Stable	Positive Global	Positive Generality	Positive Composite	Negative Internal	Negative Stable	Negative Global	Negative Generality	Negative Composite	Mean	SD	Alpha reliability
GCSE score	.28***	.27***	.13***	.23***	.27***	.05	.12***	−.15***	−.03	−.01	53.21	26.40	
Positive—internal		.59***	.32***	.51***	78***	.18***	01	−.16***	−.11***	.01	5.88	1.05	.87
Positive—stable			.52***	.85***	.86***	.01	.05	−.07*	−.02	−.01	5.17	1.02	.86
Positive—global				.89***	.78***	.03	.01	.29***	.20***	.18***	4.76	1.18	.86
Positive—generality					.94***	.03	.03	.14***	.11***	.10***	4.96	.96	.89
Positive—composite						.09**	.02	.04	.04	.08*	5.27	.87	.91
Negative—internal							.07*	.09**	.10***	57***	4.66	.87	.44
Negative—stable								.27**	.76***	.66***	4.29	.87	.65
Negative—global									.84***	.73***	3.73	1.03	.70
Negative—generality										.88***	4.02	.77	.69
Negative—composite											4.22	.62	.67

***p < .001

**p < .01

*p < .05; listwise *N* = 979.

**Table 2:** Effects of Gender, School Level, and Composite Attributional Style for Positive Events on GCSE Performance

Step	Variables	*B*	*SE*	Β	*T*	Adjusted *R* ^2^ (incremental)	*F* change (*df* 947)
1	Constant	59.07	2.57		22.99***		
	Gender	−4.47	1.72	−.08	2.60**	.007	6.77**
2	Constant	73.20	4.74		15.45***		
	Gender	1.61	1.28	.03	1.26		
	School level	−35.31	1.32	−.63	26.71***		
	PosEvents—Composite	6.75	.71	.22	9.50***	.47	423.57***
3	Constant	108.38	14.27		7.57***		
	Gender	1.60	1.27	.03	1.25		
	School level	−56.25	8.12	−1.01	6.93***		
	PosEvents—composite	.15	2.62	.01	.06		
	School × composite	3.95	1.51	.42	2.61**	.47	6.83**

***p < .001

**p < .01

*p < .05.

**Table 3:** Effects of School Level, Comparison Direction, and Composite Attributional Style for Negative Events on GCSE Performance

Step	Variables	*B*	*SE*	Β	*T*	Adjusted *R* ^2^ (incremental)	*F* change (*df* 947)
1	Constant	57.85	2.53		22.87***		
	Gender	−4.13	1.69	−.08	2.45*	.007	6.00*
2	Constant	116.21	5.09		22.85***		
	Gender	2.03	1.31	.04	1.55		
	School level	−36.87	1.36	−.66	27.03***		
	NegEvents—composite	−1.37	1.03	−.03	1.33	.47	365.17***
3	Constant	118.47	16.07		7.37***		
	Gender	2.03	1.31	.04	1.55		
	School level	−38.22	9.27	−.68	4.12***		
	NegEvents—composite	−1.90	3.73	−.04	−.51		
	School × composite	.32	2.16	.03	.15	.47	0.22

***p < .001

**p < .01

*p < .05.

### Correlational findings

GSCE scores were significantly positively correlated with making more internal (*r* = .28, *p* < .001), stable (*r* = .27, *p* < .001) and global (*r* = .13, *p* < .001) attributions for positive events, as well with the generality score (*r* = .23, *p* < .001) and the composite score (*r* = .27, *p* < .001).

GCSE scores were also significantly related to more stable attributions for negative events (*r* = .12, *p* < .001), and less‐global attributions for negative events (*r* = −.15, *p* < .001), but were unrelated to internal attributions (*r* = −.03), and unrelated to the generality score (*r* = −.03) or the composite score (*r* = −.01).

### School level and gender

Hierarchical linear regression was used to test the interaction between attributional style and school level. Gender was entered as a first block. All analyses also showed a small but significant effect of gender (β ranges from −.076 to −.085, *p*s < .05), showing that boys outperformed girls. Preliminary analyses revealed no interactions involving gender and no changes to other effects when these interaction terms were included. Therefore, interactions involving gender were not included in the model tests described below.

The analyses entered attributional style and school level in the second block and the interaction between them in the third block. In all analyses, there was a highly significant main effect of school type, (β ranges from −.63 to −.65, *p*s < .001), consistent with those shown in Tables 2 and 3. As reported earlier, students in high achieving schools outperformed those in low achieving schools.

Tables 2 and 3 provide the full regression statistics using the composite attributional style scores for negative and positive events, respectively. Interactions were probed using the MODPROBE procedure in SPSS (Hayes & Matthes, 2009).

#### Attributional style for positive events

##### Main effects of attributional style

As shown in Table 2, there was a highly significant main effect of the composite attributional style (β = .22, *t* = 9.50, *p* < .001). There were also significant main effects when we analyzed each of the three attributional dimensions separately. Participants with more internal (β = .21, *t* = 8.89, *p* < .001) stable (β = .23, *t* = 9.86, *p* < .001) or global (β = .11, *t* = 4.70, *p* < .001) attributions for positive events performed more highly. The main effect of generality was also significant (β = .19, *t* = 8.15, *p* < .001).

##### Attribution x school level

**Figure 1:**
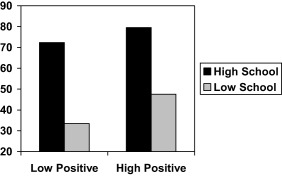
GCSE scores as a function of school level and composite attributional style for positive events. Note: Low and high positive refer to 1 SD below and above the mean composite score.

Figure 1 shows that composite attributional style for positive events interacted with school level, (β = .42, *t* = 2.61, *p* < .01). Simple slopes analyses showed that higher composite scores were associated with a greater increase in performance in the low‐achieving schools (*b* = 8.04, SE = .86, *t* = 9.31, *p* < .001) than in the high‐achieving schools (*b* = 4.10, *SE* = 1.24, *t* = 3.31, *p* < .01).

This significant interaction pattern was also obtained in separate regression analyses involving internality (β = .46, *t* = 2.81, *p* < .01), stability (β = .29, *t* = 2.14, *p* < .05), globality (β = .25, *t* = 1.99, *p* < .05), and generality (β = .33, *t* = 2.34, *p* < .05). In all of these analyses, the simple slopes tests revealed that the effects of attributional style were larger in the low achieving than in the high achieving schools.

#### Attributional style for negative events

##### Main effects of attributional style

As shown in Table 3, there was no significant main effect of the composite attributional style for negative events (β = −.03, *t* = 1.33, *ns*). When we analyzed each of the three attributional dimensions separately, participants with *less* global (*β* = −.10, *t* = 4.14, *p* < .001) attributions performed more highly. The main effects of internality (*β* = .03, *t* = 1.41, *ns*), and stability (β = .02, *t* = .78, *ns*), were not significant, but there was a significant effect of generality, (β = −.06, *t* = 2.46, *p* < .05), showing that lower generality was associated with better performance.

##### Attribution x school level

**Figure 2:**
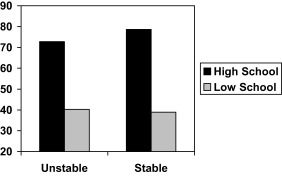
GCSE scores as a function of school level and stability attributions for negative events. Note: Unstable and stable refer to 1 SD below and above the mean stability score.

**Figure 3:**
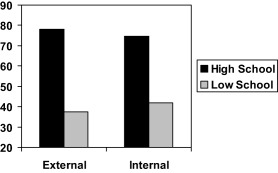
GCSE scores as a function of school level and internal attributions for negative events. Note: External and internal refer to 1 SD below and above the mean internality score.

Table 3 shows that composite attributional style for negative events did not interact significantly with school level (β = .03, *t* = .15, *ns*). Nor did globality attributions (β = −.02, *t* = .15, *ns*) or attributional generality (β = −.22, *t* = 1.45, *ns*). However, stability attribution did interact significantly with school level (β = −.36, *t* = 2.62, *p* < .01), as shown in Figure 2. Simple slopes analysis showed that stable attribution for negative events had a significant positive effect in the high‐achieving schools, (*b* = 3.31, *SE* = 1.27, *t* = 2.60, *p* < .01) but not in the low achieving schools (*b* = −.77, *SE* = .89, *t* = .86, *ns*). Moreover, internality attribution also interacted significantly with school level (β = .43, *t* = 2.82, *p* < .01), as shown in Figure 3. Simple slopes analysis showed that the effect of internality attribution was nonsignificantly negative in the high‐achieving schools, (*b* = −1.89, *SE* = 1.24, *t* = 1.52, *p* < .13) but significantly positive in the low achieving schools (*b* = 2.35, *SE* = .85, *t* = 2.76, *p* < .01).

### Ancillary analyses

Further analyses were conducted to ensure that school level—attributional style effects were not attributable to the impact of any particular school. When schools were dummy coded and included as predictors in the regression model, the interactions between attributional style and school level remained significant.

It was decided to check whether the effects on the composite attributions for positive events were independent of those for negative events. When the negative event composite was included as a covariate in the analysis (by entering it in Block 2 and/or by including its interaction with school at Block 3) the main effects and interactions reported previously for composite positive event attributions remained significant at the same levels. Parallel findings emerged when a similar analysis was conducted using generality for positive and negative events. Further details are available on request from the corresponding author.

Depressed mood was not correlated with academic achievement and when mood was included as a covariate in the regression analyses, it was not a significant predictor and there were no changes to any of the effects reported above.

## Discussion

The present study found partial support for the helplessness hypothesis derived from the reformulated model of helplessness and depression (Abramson et al., 1978). In line with the model, “healthy,” internal, stable, and global, attributional style for *positive events* was correlated with higher academic performance. This effect was stronger in the low‐achieving schools than in the high achieving schools. This is the first time the relationship between style for positive events has been evaluated in both high‐ and low‐ability contexts and the findings provide evidence that attributional style for positive events plays a more important role when the ability context is broad, than when it is selective or high achieving.

The findings in relation to attributional style for *negative events* present a different picture. Consistent with the context hypothesis, higher stable attributions for negative events were related to higher levels of performance in the high achieving schools, but not in the low achieving schools. In addition, higher levels of internality for negative events were associated with higher performance in low‐achieving schools but not in the high‐achieving schools. Higher global attributions for negative events were related to poorer performance across all schools, consistent with the helplessness hypothesis. All findings remained significant when levels of depressed mood were controlled for, providing further evidence for the independent effect of attributional style, as distinct from mood, on academic performance.

In the present study, the analysis of each attributional dimension separately, as well as the analysis of the effects of different achievement environments, provides important clarification of the relationship between attributional style and academic performance. In high achieving environments, and/or among individuals who have previously demonstrated high levels of ability, stable attributions for negative events have now been consistently demonstrated to be related to higher levels of academic performance. These effects are demonstrated in the present study, across three studies by Houston (1994) and by Gibb et al. (2002). Thus within the published literature, whenever selection on the basis of high achievement has been conducted, the effect of having a stable attributional style for negative events on subsequent performance is positive, rather than negative.

A key question is why does making stable attributions for poor achievement have a positive effect on academic performance? Stable attributions are classified as those that relate to causes which are unlikely to change over time, thus they could include personal attributes such as ability, laziness, carelessness, or other factors such as the difficulty of the subject. Unstable factors might be effort, illness, tiredness, exam room conditions, or the particular questions on an exam. The categorization of ability as a stable cause, and effort as an unstable one, dates back to Weiner (1971), but Weiner (1985) himself acknowledged that “ability may be perceived as unstable if learning is possible: effort often is perceived as a stable trait, captured with the labels lazy and industrious.” (p. 551). Dweck (1999) further differentiated between individuals with an entity perspective on ability—that intelligence is fixed and stable, and individuals with an incremental perspective—that intelligence is malleable.

Weiner (1985) stated that there was a “fundamental psychological law relating perceived causal stability to expectancy change…. If an outcome of an event is ascribed to a stable cause, then that outcome will be anticipated with increased certainty or with an increased expectancy in the future.” This “fundamental” law may indeed hold. What appears to be more ambiguous, are the consequences of this expectancy. It can be argued that attribution of unstable causes for failure (tiredness, a bad exam paper) might be categorized as “excuses,” which may not give rise to any change of behavior on an individual's part. These attributions may allow the individual to believe that the outcome will be different the next time. In the case of academic performance, repeated recourse to such excuses is unlikely to promote improvements in performance. Recognizing that one finds mathematics difficult, or that one attends insufficiently to detail, is an important step toward taking remedial action and ensuring that the outcome is different next time. Indeed, the present findings are consistent with the idea that those who accomplish a higher level of achievement are those who have learned that poor outcomes require action, not excuses. Taking responsibility for change is critical to increasing achievement.

The notion of “taking responsibility” may also explain the findings in relation to internal attributions for negative events. In this study, internal attributions for negative events were associated with higher performance in the low‐achieving schools. One explanation may be that, in an environment in which high levels of performance are not generally expected, taking responsibility for one's own failure may lead to action to avoid future failure.

### Strengths, limitations, and implications

The present research demonstrates that the relationship between attributional style and academic performance varies according to academic ability and/or achievement context. In high‐achieving schools, students with a stable style of attribution for negative events outperform those with an unstable style. In low‐achieving schools, students with an internal style for negative events perform better in examinations than those with an external style. These findings are consistent across individual schools in each category and thus cannot be an artefact of the type of teaching or culture within a particular school. This is a challenge to traditional theories of the impact of attribution on performance (e.g., Weiner, 1985) and has implications for attributional retraining (Boese, Stewart, Perry, & Hamm, 2013; Morris, 2013). Attribution theory and attributional retraining have had a significant impact on interventions aimed to improve academic achievement since the 1970s (Chodkiewicz & Boyle, 2014). The present research shows clearly that having stable and internal styles of attribution can be related to better performance in some achievement contexts and thus interventions designed to change attributions for failure to those which are external and unstable may be fundamentally flawed. While blaming failure on lack of ability may be maladaptive, attribution to some stable and internal causes clearly has a positive effect on academic performance. Thus generalizations about the positive effect of broad categories of attributions on performance should be avoided. Instead the focus for interventions on how cognitions influence motivation and learning should be placed on expectancies that promote “taking responsibility.”

The present research has provided a comprehensive test of the predicted relationship between different dimensions of attributional style and academic performance, and the moderating effect of achievement context. A key strength of the research is that it has employed a sufficiently large and diverse sample to examine achievement context in detail and to provide confidence in the implications of the findings. The limitations for the work are that it only employed samples from the English high school education system; cross cultural replications could cast further light on the role of achievement context. In addition, the current study examined children aged 15–16 years, future research could focus on possible developmental differences in the attribution–performance relationship and the achievement context at an earlier phase in school education.

It can be concluded that the present research presents important qualifications to theories of the relationship between attribution and academic performance; stable and internal attributional style *can* lead to better academic performance. This means that the role of attributional retraining in improving academic performance should emphasize attributions that give rise to responsibility and action, rather than focusing on broad categories of attributional dimension.
